# Left Atrial Appendage Closure Yields Favorable Cardio- and Cerebrovascular Outcomes in Patients With Non-valvular Atrial Fibrillation and Prior Stroke

**DOI:** 10.3389/fneur.2021.784557

**Published:** 2022-01-10

**Authors:** Mingzhong Zhao, Mengxi Zhao, Cody R. Hou, Felix Post, Nora Herold, Jens Walsleben, Zhaohui Meng, Jiangtao Yu

**Affiliations:** ^1^Department of Cardiology, Helmut-G-Walther-Klinikum, Lichtenfels, Germany; ^2^Heart Center, Zhengzhou Ninth People's Hospital, Zhengzhou, China; ^3^Department of Neurology, Beijing Tiantan Hospital, Capital Medical University, Beijing, China; ^4^Cardiovascular Division, Department of Medicine, University of Minnesota Medical School, Minneapolis, MN, United States; ^5^Clinic for General Internal Medicine and Cardiology, Catholic Medical Center Koblenz-Montabaur, Koblenz, Germany; ^6^Department of Cardiology, Kunming Medical University, Kunming, China

**Keywords:** atrial fibrillation, left atrial appendage closure, stroke, thromboembolism, bleeding, anticoagulants

## Abstract

**Introduction:** Patients with non-valvular atrial fibrillation (NVAF) and previous stroke are at significantly higher risk of stroke recurrence. Data on the efficacy of left atrial appendage closure (LAAC) on these patients is limited. The aim of this study was to investigate the differences of LAAC efficacy on long-term cardio- and cerebrovascular outcomes in NVAF patients with vs. without prior stroke.

**Methods:** Three hundred and seventy consecutive NVAF patients who underwent LAAC were enrolled and divided into stroke and non-stroke groups based on history of previous stroke. Endpoints, such as thromboembolism, major bleeding, and mortality post-LAAC, were followed up among groups.

**Results:** Patients in the stroke group had higher mean CHA_2_DS_2_-VASc and HAS-BLED scores compared to the non-stroke group (5.1 vs. 3.6 and 4.1 vs. 3.4, both P < 0.001, respectively). Over a median follow-up of 2.2 years, there were no significant differences in incidence rates of thromboembolism, device-related thrombus (DRT), major bleeding, and combined efficacy endpoints between the two groups. In both stroke and non-stroke groups, LAAC decreased the risk of thromboembolism [relative risk reduction (RRR) 87.5%, P = 0.034, and 74.6%, P = 0.004, respectively] and major bleeding (RRR 68.8%, P = 0.034, and 68.6%, P = 0.007, respectively) compared with predicted risk. The RRR in thromboembolism was greater in patients with vs. without prior stroke (OR 2.45, 95% CI: 1.20–5.12, P = 0.016). The incidence rates of all-cause mortality and non-cardiovascular death were similar between the two groups, but the risks of cardiovascular death post-LAAC both before (1.4% vs. 8.1%, respectively, P = 0.038) and after adjustment for confounding factors (P = 0.048) were significantly decreased in the stroke group.

**Conclusions:** Patients with vs. without prior stroke did not exhibit a worse clinical prognosis after LAAC. LAAC may provide an increased benefit in cardio-cerebrovascular outcomes in patients with previous stroke compared to those without previous stroke. Further research is necessary to evaluate the efficacy of LAAC in this field.

## Introduction

Atrial fibrillation (AF) is a major global health burden. In the United States, AF prevalence is rising, with a projected 12.1 million patients by 2030 (1). In the European Union, AF patients were estimated to be 7.6 million in 2016 and are projected to increase to 14.4 million by 2060 (2). For patients with non-valvular AF (NVAF), which account for a majority of AF patients, there is a 5-fold increase in the incidence of cardiogenic stroke and 2-fold increase in the risk of all-cause mortality compared with patients without AF (3). Thromboembolism secondary to NVAF, including ischemic stroke, transient ischemic attack (TIA), and systemic embolism, is a devastating event in these patients. However, these complications can be prevented.

Oral anticoagulant therapy is the cornerstone of cardioembolic stroke prevention in AF patients (4). Both traditional oral warfarin and non–vitamin K antagonist oral anticoagulant (NOACs) therapies may reduce the risk of thromboembolism, but NOACs demonstrated lower rates of stroke/systemic embolism or bleeding risk and better health-related quality of life over oral warfarin (5, 6). Oral anticoagulants still face limitations in clinical practice. Even NOACs encounter issues with bleeding and medication compliance attributed to higher payments and higher rate of discontinuation in the first year (7). In recent years, percutaneous left atrial appendage closure (LAAC) has been deemed as an alternative treatment for stroke prevention in NVAF patients. Long-term follow-up demonstrated that LAAC provided cardioembolic stroke prevention in NVAF comparable to warfarin, with additional reductions in hemorrhagic stroke and mortality (8). Furthermore, LAAC also proved to be noninferior to NOACs in stroke prevention efficacy and was associated with lower risk of major bleeding and all-cause mortality (9, 10).

AF patients with a history of prior stroke, TIA or systemic embolism are a fragile patient group. Some subgroups such as those with older age, fresh ischemic lesions, or chronic kidney disease present with an increased risk of disability, bleeding (particularly intracranial hemorrhage), or mortality under oral anticoagulation (4, 11). These high-risk subgroups may be good candidates for LAAC. A recent small-sample study reported on the effectiveness of LAAC in recurrent stroke and bleeding risk reduction in AF patients with previous stroke (12). However, data comparing LAAC efficacy in the risk reduction of thromboembolism, major bleeding, and mortality between patients with prior stroke and those without are sparse. The aim of the study was to evaluate the influence of LAAC on long-term outcomes in such special patient subgroups.

## Methods

### Study Population

Consecutive NVAF patients who underwent percutaneous LAAC with the WATCHMAN® device (Boston Scientific, Marlborough, MA, USA) at Helmut-G.-Walther Klinikum, Lichtenfels, Germany were enrolled in an observational registry between February 2012 and June 2018. The major inclusion criteria for LAAC procedure were patients with high risk of cardioembolism and/or contraindication for long-term anticoagulation therapy, or unwillingness to take anticoagulation drugs. Those who suffered from malignant tumor or end stage diseases with life expectancy shorter than 1 year or presented with thrombus in the left atria/left atrial appendage were excluded. The study protocol complies with the Declaration of Helsinki and was approved by the Ethics Committee at Helmut-G.-Walther Klinikum, Lichtenfels, Germany. All patients provided written informed consent for the device implantation. The cohort was divided into two groups: stroke group (patients with a history of prior stroke) and non-stroke group (patients without a history of prior stroke). An analysis of demographic and clinical characteristics, peri-procedural data, and long-term outcomes was performed for all patients.

### LAAC Procedure

The LAAC procedure was previously described in literature (13). Briefly, the procedure was performed under general anesthesia and guided by intra-procedural transesophageal echocardiography (TEE) and fluoroscopy. The WATCHMAN® device was implanted via an atrial septum puncture and use of a delivery system according to the device's directions for use. TEE and X-ray angiograms were used to guide device size selection based on the recommended compression ratio in relation to the size of left atrial appendage (LAA). The device was released after the device's implantation met PASS criteria (position, anchor, size, and seal). After implantation, patients took antithrombotic drugs to allow time for device endothelialization: (1)warfarin or NOACs combined with aspirin, or low molecular weight heparin (LMWH), such as enoxaparin plus aspirin, or aspirin plus clopidogrel if subjects were contraindicated to anticoagulants, was administered during hospitalization; (2) during the period from discharge to 45 days post procedure, all patients kept on the previous antithrombotic strategies except from those treated with subcutaneous enoxaparin injection, whose treatment regimen was switched to oral anticoagulants or antiplatelets. (3) at approximately 45 days post procedure, if TEE imaging showed adequate closure of LAA [no residual peri-device jet >5mm in width and no device-related thrombus (DRT)], all anticoagulants were discontinued and clopidogrel combined with aspirin was administered until 6 months post procedure; (4) patients were prescribed aspirin alone indefinitely after the 6-month visit following TEE indicating adequate closure. If inadequate closure was observed or a thrombus was detected by TEE, warfarin or NOACs combined with aspirin was restarted until an adequate seal or complete resolution of the thrombus was confirmed by repeat TEE exam.

### Endpoints

For each participant, TEE follow-up visit was scheduled at 45 days and 6 months after the procedure. The clinical follow-up times were synchronized with TEE follow-up schedule, with an additional final visit at the end of the study. Implant success was defined as an adequate closure of the LAA by the occluder without residual peri-device flow >5 mm in width and DRT.

Implant success, procedural data, antithrombotic regimen, and peri-procedural complications within 7 days were recorded. The prognostic endpoints of long-term follow-up included thromboembolism (ischemic stroke, TIA, and systemic embolism), major bleeding (cerebral hemorrhage, gastrointestinal bleeding, and other major bleeding), DRT, and all-cause death (cardiovascular death, and non-cardiovascular death). Combined efficacy endpoints consisted of thromboembolism and all-cause death.

### Assessment of Thromboembolic and Bleeding Risks

The observed annual rate of thromboembolic or major bleeding events was expressed as events per 100 patient-years, respectively, which was calculated as the total number of patients with thromboembolic or major bleeding events in a cohort divided by the total patient-years of follow-up and then multiplied by 100. The expected annual rate of thromboembolic or major bleeding events was calculated as the mean of each individual annual risk in a cohort, based on the CHA_2_DS_2_-VASc and HAS-BLED scores, respectively (14, 15). Thromboembolism or major bleeding relative risk reduction (RRR) was calculated as follows: (expected rate – observed rate) / expected rate.

### Statistical Analysis

Categorical variables are presented as counts and percentages. Continuous variables were checked for normal distribution using the Shapiro-Wilk test. Continuous variables are presented as mean ± standard deviation (SD) or median (interquartile range Q1–25th percentile and Q3–75th percentile). χ^2^ tests were used to compare the differences of event rates between groups. Student's *t*-tests (for normal distribution) or Mann-Whitney U tests (for non-normal distribution) were used to compare differences of continuous variables. To investigate the efficacy of LAAC on reductions of thromboembolic and major bleeding risks, the comparison of observed annual rate of thromboembolic or major bleeding events and predicted risk was analyzed by using a χ^2^ test, with odds ratio (OR) and their 95% confidence intervals (CI). The number needed to treat (NNT) to prevent one thromboembolic or major bleeding event by LAAC was calculated in overall cohort, stroke group, and non-stroke group as NNT = 1 / {predicted annual rate of event—observed annual rate of event}. To accurately evaluate the impact of LAAC on mortality, a propensity-score match (PSM) analysis at a 1:1 ratio using a caliper width equal to 0.2 of the standardized mean difference of the logit without replacement among the two groups was used to reduce the effect of potential confounding factors which included 12 clinically relevant variables, such as age, sex, coronary heart disease (CHD), diabetes, hypertension, chronic heart failure, previous major bleeding, abnormal liver function, impaired renal function, CHA_2_DS_2_-VASc score, HAS-BLED score, and types of atrial fibrillation. Kaplan-Meier survival curves were used to assess the cumulative ratio of freedom from mortality events, and the differences in mortality between the two groups were compared with Log-rank tests. All significance tests were 2-tailed, and a *p* < 0.05 was considered significant. All analyses were performed with SPSS version 26.0 (SPSS Inc., Chicago, Illinois).

## Results

### Baseline Characteristics of the Study Cohort

Out of the 379 patients with NVAF (stroke group: 76 cases; non-stroke group: 303 cases), 370 (97.6%) patients with successful device implantation were included in the study (74 cases in stroke group and 296 cases in non-stroke group), excluding nine patients in which LAAC procedures were halted because of unsuitable LAA anatomy in six patients, cardiac tamponade in two patients, and repeated DRT in one patient. No significant difference was found in implant success between the two groups (97.4% vs. 97.7%, P = 1.000). Among the 74 patients suffered a prior stroke, 66 cases (58 cases for ischemic stroke, eight cases for hemorrhagic stroke) had stroke onset >6 months, six cases were within 3–6 months of ischemic stroke onset, and only two cases were within 6 weeks of ischemic stroke onset.

Among the two groups, baseline demographic and clinical characteristics were comparable except for CHA_2_DS_2_-VASc score and HAS-BLED score, which were significantly higher in the stroke group (both P < *0.001*) (Table 1).

**Table 1:** Patient baseline characteristics.

**Variables**	**Overall**	**Stroke**	**Non-stroke**	***P* value**
	***n* = 370**	***n* = 74**	***n* = 296**	
Age, years (mean ±SD)	75.1 ± 7.8	76.1 ± 8.2	74.8 ± 7.7	0.211
≥75 years, *n* (%)	221 (59.7)	48(64.9)	173 (58.5)	0.314
Male, *n* (%)	248 (67.0)	46 (62.1)	202 (68.2)	0.320
Hypertension, *n* (%)	298 (80.5)	65 (87.8)	233 (78.1)	0.100
Diabetes mellitus, *n* (%)	103 (27.3)	21 (28.4)	82 (27.7)	0.908
CHD, *n* (%)	181 (49.2)	30 (40.5)	151 (51.0)	0.107
Chronic heart failure^▴^, *n* (%)	62 (16.8)	12 (16.2)	50 (16.9)	0.889
Previous major bleeding, *n* (%)	132 (35.7)	31 (41.9)	101 (34.1)	0.212
Abnormal liver function^⋆^, *n* (%)	49 (13.2)	7 (9.5)	42 (14.2)	0.283
Impaired renal function^♦^, *n* (%)	169 (45.7)	33 (44.6)	136 (46.0)	0.835
CHA_2_DS_2_-VASc score (mean ±SD)	3.9 ± 1.5	5.1 ± 1.6	3.6 ± 1.3	<0.001
HAS-BLED score (mean ±SD)	3.5 ± 1.0	4.1 ± 1.0	3.4 ± 1.0	<0.001
AF, paroxysmal, *n* (%)	127 (34.3)	22 (29.7)	105 (35.5)	0.352
AF, persistent or permanent, *n* (%)	243 (65.7)	52 (70.3)	191 (64.5)	0.352

*Categorical variables are expressed as frequencies (n) and percentages (%). Continuous data are reported as means and standard deviation or median and interquartile range*.

▴ *defined as presence of left ventricular ejection fraction (LVEF) <40% or symptomatic heart failure;*

⋆ *defined as a prior liver disease or presence of elevated liver enzymes (alanine aminotransferase/aspartate aminotransferase ≥ 2 × upper limit of normal) at admission;*

♦ *defined as an estimated glomerular filtration rate (eGFR) <60 ml/min per 1.73 m^2^*.

*CHD, coronary heart disease; AF, atrial fibrillation*.

### Procedural Data and Postprocedural Antithrombotic Regimen

No significant differences were observed in LAA width and depth, device size, proportion of patients with peri-device flow, and fluoroscopy time between the two groups. However, X ray-dose and contrast volume were increased significantly in the non-stroke group compared with the stroke group (Table 2).

**Table 2:** Procedural data and periprocedural antithrombotic regimen.

**Variables**	**Overall**	**Stroke**	**Non-stroke**	***P* value**
	***n =* 370**	***n =* 74**	***n =* 296**	
LAA width, mm (Mean ± SD)	20.2 ± 3.4	20.0 ± 3.6	20.2 ± 3.9	0.705
LAA depth, mm (Mean ± SD)	27.1 ± 4.7	29.3 ± 5.7	26.6 ± 5.1	0.115
Device size, mm (Mean ± SD)	25.2 ± 3.2	25.4 ± 3.1	25.1 ± 3.1	0.407
Peri-device flow, *n* (%)	10 (2.7)	2 (2.7)	8 (2.7)	1.000
<3 mm	9	2	7	
3–5 mm	1	0	1	
>5 mm	0	0	0	
Fluoroscopy time (min), median (IQR)	8.4 (6.2; 12.1)	7.6 (6.1; 10.4)	8.6 (6.2; 12.9)	0.184
X ray-dose (mGy*cm^2^), median (IQR)	5,056 (3,112; 8,616)	3,622 (2,759; 6,545)	5,326 (3,206; 8,863)	<0.001
Contrast (ml), median (IQR)	90 (70; 110)	80 (60;100)	90 (70; 110)	0.015
Antithrombotic therapy postprocedure during hospitalization				
Warfarin, *n* (%)	2 (0.5)	0 (0)	2 (0.7)	1.000
Aspirin + warfarin, *n* (%)	40 (10.8)	7 (9.5)	33 (11.2)	0.676
Aspirin + LMWH, *n* (%)	236 (63.8)	37 (50.0)	199 (67.2)	0.006
Aspirin + NOACs, *n* (%)	40 (10.8)	12 (16.2)	28 (9.6)	0.094
Aspirin + clopidogrel, *n* (%)	48 (13.0)	16 (21.6)	32 (10.8)	0.013
None, *n* (%)	4 (1.1)	2 (2.7)	2 (0.7)	0.180

*Categorical variables are expressed as frequencies (n) and percentages (%). Continuous data are reported as means and standard deviation or median and interquartile range*.

*LAA, left atrial appendage; IQR, interquartile range; LMWH, low molecular weight heparin; NOACs, non-vitamin K antagonist oral anticoagulants*.

During the early stage post procedure (during hospitalization), the stroke group was more frequently prescribed aspirin plus clopidogrel (*P* = 0.013) and less frequently prescribed aspirin plus LMWH therapy (*P* = 0.006) than the non-stroke group. No statistical differences were found for the remaining antithrombotic regimen between groups (Table 2).

### Peri-Procedural Complications Within 7 Days

There were no significant differences in peri-procedural complications within 7 days between the two groups (Table 3).

**Table 3:** Peri-procedural complications within 7 days.

**Variables**	**Overall**	**Stroke**	**Non-stroke**	***P* value**
	***n =* 370**	***n =* 74**	***n =* 296**	
Stroke, *n* (%)	1 (0.3)	0 (0)	1 (0.3)	1.000
TIA, *n* (%)	0 (0)	0 (0)	0 (0)	1.000
Other systemic embolism, *n* (%)	0 (0)	0 (0)	0 (0)	1.000
Major bleeding, *n* (%)	2 (0.5)	0 (0)	2 (0.7)	1.000
Pericardial effusion/cardiac tamponade, *n* (%)	3 (0.8)	1 (1.4)	2 (0.7)	0.489
Severe vascular complication, *n* (%)	4 (1.1)	1 (1.4)	3 (1.0)	1.000
Device-related death, *n* (%)	0 (0)	0 (0)	0 (0)	1.000
Total, *n* (%)	10 (2.7)	2 (2.7)	8 (2.7)	1.000

*Categorical variables are expressed as frequencies (n) and percentages (%)*.

*TIA, transient ischemic attack*.

### Long-Term Outcomes

In this cohort, median follow-up was 2.2 years, which resulted in 831 patient-years of follow-up, with 161 patient-years in the stroke group and 671 patient-years in the non-stroke group, respectively. There were no significant differences in average follow-up time and TEE visit rate between the two groups (Table 4).

**Table 4:** Outcomes of long-term follow-up.

**Adverse events**	**Overall**	**Stroke**	**Non-stroke**	***P* value**
	***n =* 370**	***n =* 74**	***n =* 296**	
Follow-up time, (days)	820.2 ±	792.8 ±	827.0 ±	0.629
(mean ± standard deviation)	544.5	595.5	531.9	
Follow-up-TEE, *n* (%)	370 (100)	74 (100)	296 (100)	1.000
Thromboembolism, *n* (%)	13 (3.5)	2 (2.7)	11 (3.7)	0.672
Ischemic stroke, *n* (%)	8 (2.2)	1 (1.4)	7 (2.4)	1.000
TIA, *n* (%)	5 (1.4)	1 (1.4)	4 (1.4)	1.000
Systemic embolism, *n* (%)	0 (0)	0 (0)	0 (0)	1.000
DRT, *n* (%)	20 (5.4)	4 (5.4)	16 (5.4)	1.000
Major bleeding, *n* (%)	19 (5.1)	4 (5.4)	15 (5.1)	0.906
Cerebral hemorrhage, *n* (%)	3 (0.8)	0 (0.0)	2 (0.7)	1.000
GI bleeding, *n* (%)	14 (3.8)	3 (4.1)	12 (4.1)	1.000
Other major bleeding, *n* (%)	2 (0.5)	1 (1.4)	1 (0.3)	0.360
All-cause death, *n* (%)	56 (15.1)	10 (13.5)	46 (15.5)	0.663
Cardiovascular death, *n* (%)	25 (6.8)	1 (1.4)	24 (8.1)	0.038
Non-cardiovascular death, *n* (%)	31 (8.4)	9 (12.2)	22 (7.4)	0.189
Combined efficacy endpoints, *n* (%)	67 (18.1)	12 (16.2)	55 (18.6)	0.637

*Categorical variables are expressed as frequencies (n) and percentages (%). Continuous data are reported as means and standard deviation*.

*TEE, transesophageal echocardiography; TIA, transient ischemic attack; DRT, device-related thrombus; GI, Gastrointestinal*.

**Figure 1:**
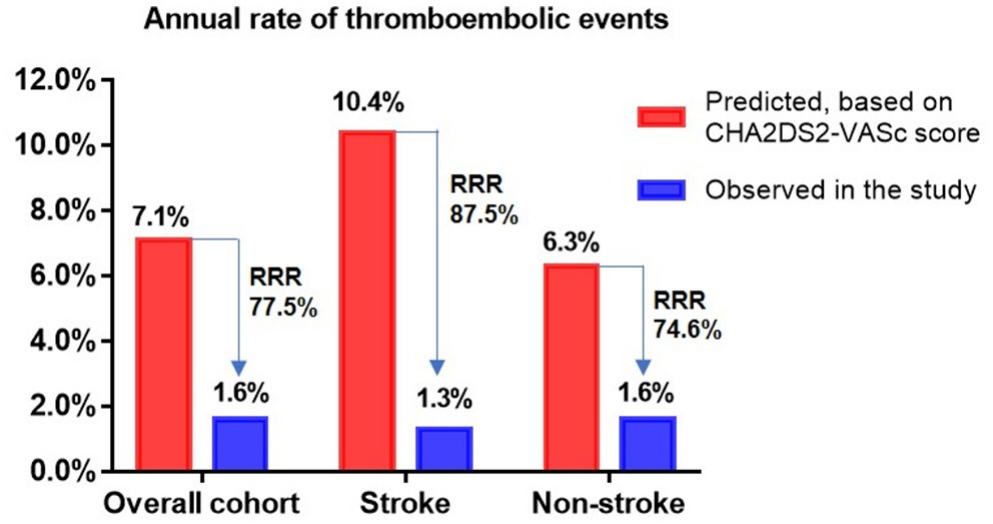
Effectiveness of LAAC in reducing thromboembolic risk in different groups. RRR, relative risk reduction.

**Figure 2:**
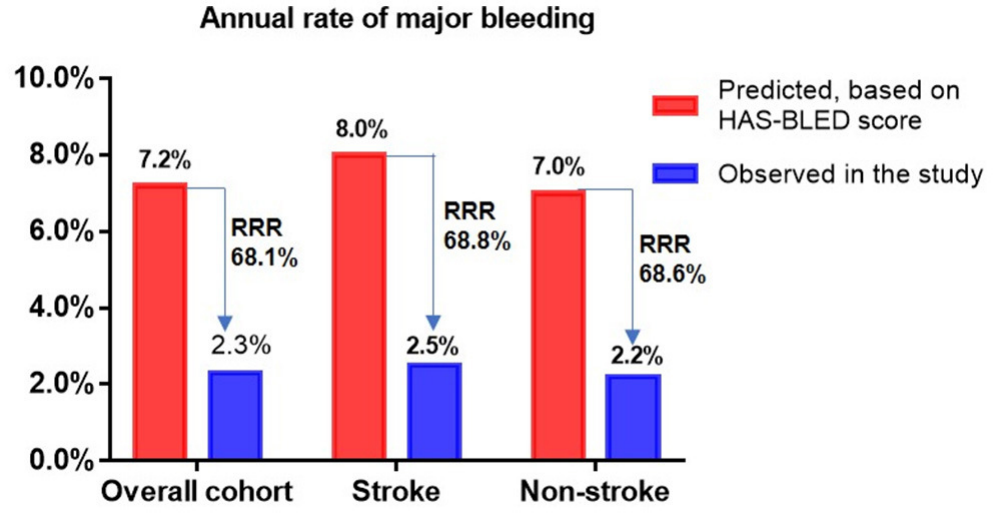
Effectiveness of LAAC in reducing major bleeding risk in different groups. RRR, relative risk reduction.

The incidence rates of thromboembolism (2.7 vs. 3.75%, P = 0.672) and major bleeding (5.4 vs. 5.1%, *P* = 0.906) were similar between the two groups (Table 4). Taking into account patient follow-up time, the observed annual rates of thromboembolism in the overall cohort, stroke group, and non-stroke group were 1.6, 1.3, and 1.6%, respectively, whereas the expected annual rates based on CHA_2_DS_2_-VASc score were 7.1, 10.4, and 6.3%, respectively. This corresponded to a 77.5, 87.5, and 74.6% RRR for thromboembolic events in the overall cohort (OR: 4.59, 95% CI: 1.97–10.59, P <0.001), stroke group (OR: 8.85, 95% CI: 1.28–99.43, *P* = 0.034) and non-stroke group (OR: 3.99, 95% CI: 1.56–9.89, *P* = 0.004) according to Kaplan-Meier estimation, with NNT values for LAAC to prevent one thromboembolic event being 18, 11, and 21 over the follow-up period, respectively. The RRR in thromboembolic events was greater in patients with prior stroke vs. those without prior stroke (OR 2.45, 95% CI: 1.20–5.12, *P* = 0.016) (Figure 1). Meanwhile, the observed annual rates of major bleeding were 2.3, 2.5, and 2.2% in the overall cohort, stroke group, and non-stroke group, respectively, whereas the expected annual rates based on HAS-BLED score were 7.2, 8.0, and 7.0%, respectively. This constituted a RRR of 68.1, 68.8, and 68.6% for major bleeding events in the overall cohort (OR: 3.16, 95% CI: 1.52–6.83, P = 0.002), stroke group (OR: 7.63, 95% CI: 1.28 — 86.95, P = 0.034) and non-stroke group (OR: 3.15, 95% CI: 1.36 — 7.94, P = 0.007), with NNT values for LAAC to prevent one major bleeding event being 20, 18, and 21 over the follow-up period, respectively. The percentage of RRR in major bleeding was comparable between the stroke and non-stroke groups (OR 1.02, 95% CI: 0.58 — 1.77, P = 0.955) (Figure 2).

**Figure 3:**
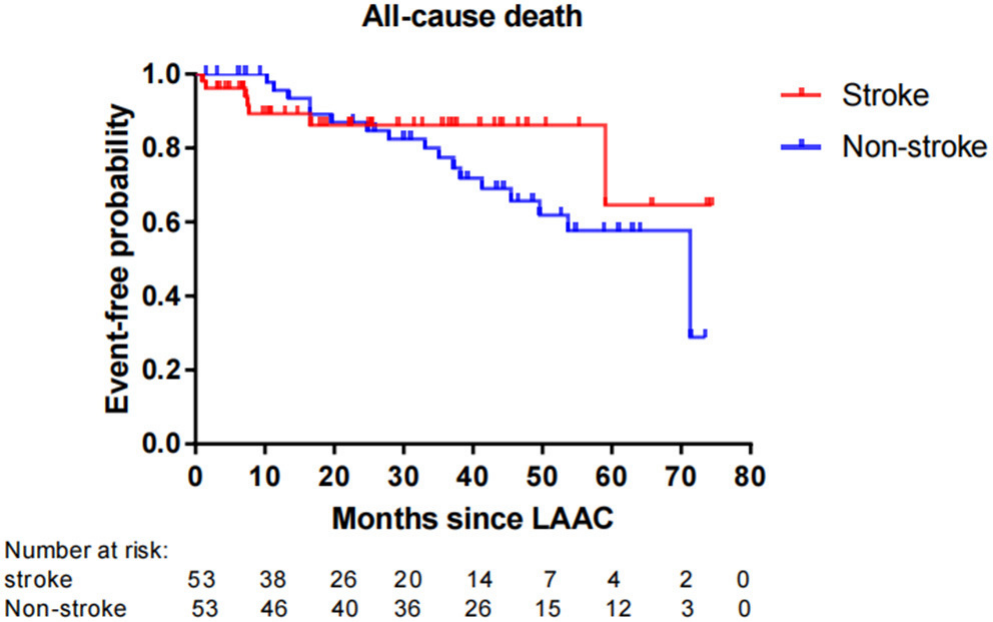
Cumulative ratio of freedom from all-cause death associated with LAAC in the PSM cohort. The number of AF patients at risk are presented along the time axis. LAAC, left atrial appendage closure.

**Figure 4:**
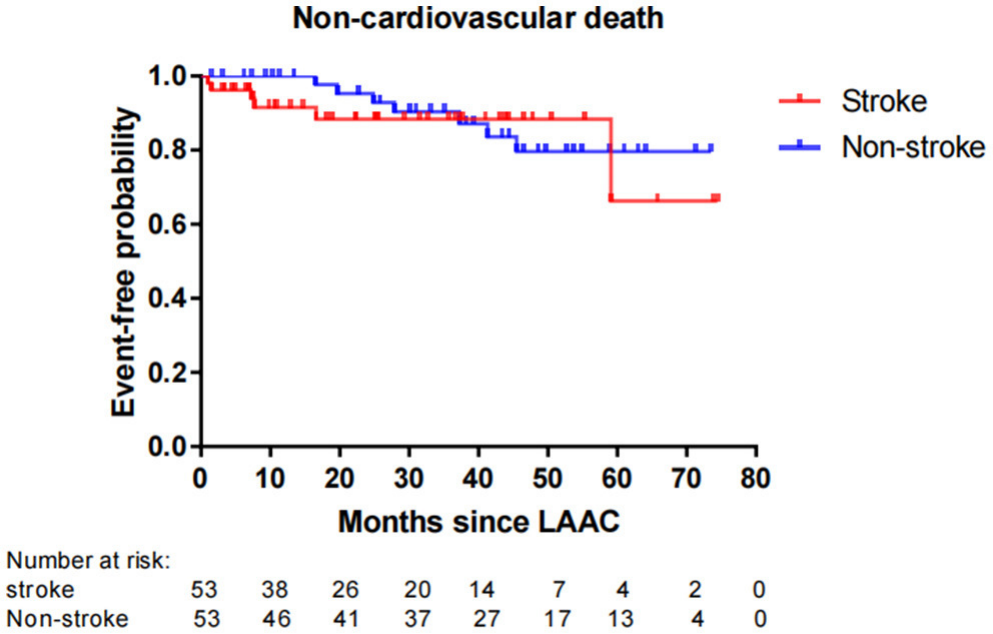
Cumulative ratio of freedom from non-cardiovascular death associated with LAAC in the PSM cohort. The number of AF patients at risk are presented along the time axis. LAAC, left atrial appendage closure.

**Figure 5:**
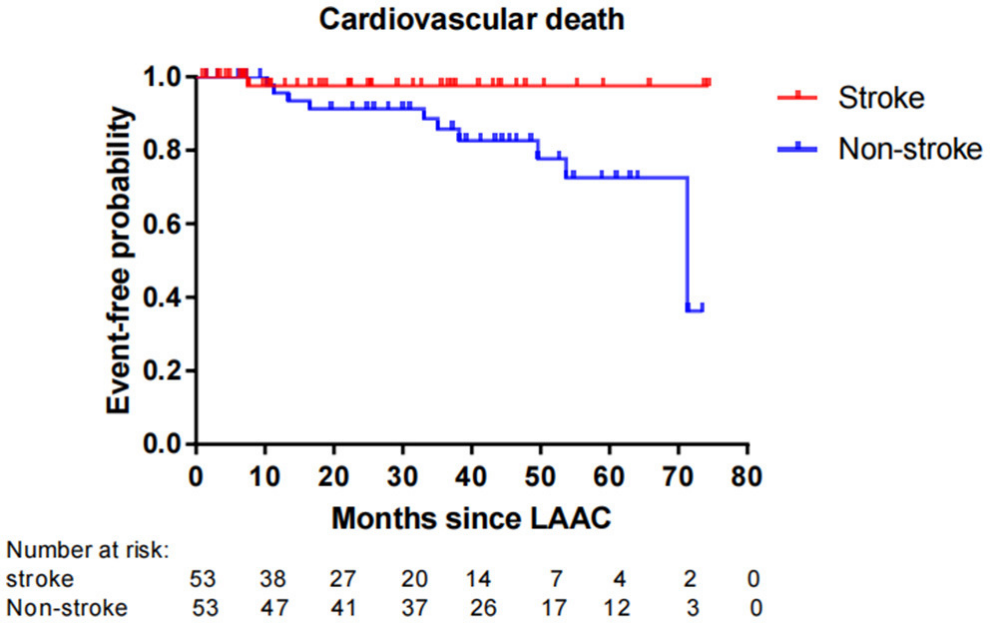
Cumulative ratio of freedom from cardiovascular death associated with LAAC in the PSM cohort. The number of AF patients at risk are presented along the time axis. LAAC, left atrial appendage closure.

Fifty-six patients (15.1%) died during long-term follow-up, with 25 cases (6.8%) from cardiovascular causes and 31 (8.4%) from non-cardiovascular causes. There were no significant differences in all-cause death (*P* = 0.663) and non-cardiovascular death (*P* = 0.189) between the stroke and non-stroke groups. However, the incidence rate of cardiovascular death was significantly lower in the stroke group than non-stroke group (1.4 vs. 8.1%, *P* = 0.038) (Table 4). After performing PSM, 106 out of 370 patients were selected as the PSM cohort, with 53 cases in stroke group and 53 cases in non-stroke group. The baseline characteristics and postprocedural antithrombotic regimen of the PSM cohort are presented in Table 5. In the PSM cohort, seven patients died in the stroke group, with one case of cardiovascular death and six cases of non-cardiovascular death; 17 deaths occurred in non-stroke group, with 10 cases of cardiovascular death and seven cases of non-cardiovascular death. Kaplan–Meier survival curves analysis demonstrated that the cumulative ratio of freedom from all-cause death (*P* = 0.340) (Figure 3) or non-cardiovascular death (P = 0.610) (Figure 4) was similar between the two groups, but the cumulative ratio of freedom from cardiovascular death was significantly higher in the stroke group (*P* = 0.048) (Figure 5).

**Table 5:** The comparisons of baseline characteristics and postprocedural antithrombotic regimen in the propensity-score matching cohort.

**Variables**	**Overall**	**Stroke**	**Non-stroke**	**P value**
	***n =* 106**	***n =* 53**	***n =* 53**	
Age, years (mean ± SD)	75.4 ± 7.8	75.1 ± 9.1	75.7 ± 6.4	0.703
≥75 years, *n* (%)	68(64.2)	31 (58.5)	37 (69.8)	0.224
Male, n (%)	78 (73.6)	438(71.6)	40(75.4)	0.660
Hypertension, *n* (%)	88 (83.0)	46 (86.7)	42 (79.2)	0.301
Diabetes mellitus, *n* (%)	28 (26.4)	13 (24.5)	15 (28.3)	0.660
CHD, *n* (%)	49 (46.2)	22 (41.5)	27 (50.9)	0.330
Chronic heart failure^▴^, *n* (%)	18 (16.9)	9 (16.9)	9 (16.9)	1.000
Previous major bleeding, *n* (%)	41 (38.6)	24 (45.2)	17 (32.0)	0.163
Abnormal liver function^⋆^, *n* (%)	9 (8.4)	7 (13.2)	2 (3.7)	0.161
Impaired renal function^♦^, *n* (%)	50 (47.1)	24 (45.2)	26 (49.0)	0.697
CHA_2_DS_2_-VASc score (mean ± SD)	4.5 ± 1.3	4.6 ± 1.4	4.4 ± 1.2	0.413
HAS-BLED score (mean ± SD)	3.9 ± 1.0	4.0 ± 1.0	3.8 ± 1.0	0.257
AF, paroxysmal, *n* (%)	30 (28.3)	17 (32.1)	13 (24.5)	0.388
AF, persistent or permanent, *n* (%)	76 (71.7)	36 (67.9)	40 (75.5)	0.388
Antithrombotic therapy postprocedure during hospitalization				
Warfarin, *n* (%)	1 (0.9)	1 (1.9)	0 (0)	1.000
Aspirin + warfarin, *n* (%)	8 (7.6)	5 (9.4)	3 (5.7)	0.716
Aspirin + LMWH, *n* (%)	75 (20.3)	35 (66.0)	40 (75.5)	0.286
Aspirin + NOACs, *n* (%)	6 (5.7)	3 (5.7)	3 (5.7)	1.000
Aspirin + clopidogrel, *n* (%)	15 (14.2)	9 (17.0)	6 (11.3)	0.403
None, *n* (%)	1 (0.9)	0 (0)	1 (1.9)	1.000

*Categorical variables are expressed as frequencies (n) and percentages (%). Continuous data are reported as means and standard deviation or median and interquartile range*.

▴ *defined as presence of left ventricular ejection fraction (LVEF) <40% or symptomatic heart failure;*

⋆ *defined as a prior liver disease or presence of elevated liver enzymes (alanine aminotransferase/aspartate aminotransferase ≥ 2 × upper limit of normal) at admission;*

♦ *defined as an estimated glomerular filtration rate (eGFR) <60 ml/min per 1.73 m^2^.*

*CHD, coronary heart disease; AF, atrial fibrillation; LMWH, low molecular weight heparin; NOACs, non-vitamin K antagonist oral anticoagulants*.

No significant difference for the incidence of DRT (P = 1.000) or combined efficacy endpoints (*P* = 0.637) was found between the stroke and non-stroke groups (Table 4).

## Discussion

The present study revealed four major findings. First, although NVAF patients with prior stroke had higher CHA_2_DS_2_-VASc and HAS-BLED scores, the ostium diameter and depth of left atrial appendage, implant success rate, and the incidences of periprocedural complications were comparable in comparison to those without prior stroke. Second, long-term thromboembolism and major bleeding risks following LAAC did not differ between the stroke and non-stroke groups. Third, the observed annual rates of thromboembolic and major bleeding events after LAAC were significantly lower than the predicted risks according to CHA_2_DS_2_-VASc score and HAS-BLED score in both the overall cohort and subgroups respectively. Patients with prior stroke experienced a more favorable RRR of recurrent thromboembolic events post LAAC than those without prior stroke. Fourth, the risks of cardiovascular death post LAAC both before and after adjustment for confounding factors were significantly decreased in the stroke group over the non-stroke group.

Patients with AF and a history of prior stroke often had higher CHA_2_DS_2_-VASc and HAS-BLED scores and were usually considered to be at high risk of recurrent ischemic stroke (16, 17). Although patients in the stroke group were also deemed as a high risk population, LAA width and depth, device size, implant success, and peri-procedural complications within 7 days were still comparable between the two groups.

Regarding the differences in thromboembolic and major bleeding risks between AF patients with and without previous stroke, previous studies showed that recurrent stroke occurred more frequently in AF patients with prior stroke *vs*. those without, even on anticoagulation (18, 19). Kodani et al. also reported that AF patients with secondary stroke prevention experienced significantly increased risks of thromboembolism and major bleeding after warfarin treatment compared to those with primary stroke prevention (20). But what about the effects of LAAC in these subgroups? For the stroke group of our study, despite having a higher prescription rate of aspirin plus clopidogrel but a lower prescription rate of combination of aspirin and LMWH post procedure during hospitalization, the incidence rates of thromboembolism and major bleeding during long-term follow-up were as low as 2.7 and 5.4%, respectively, and the event rates were almost identical to those in the non-stroke group. The rate of thromboembolism in the stroke group post LAAC was lower than that in patients with cardioembolic stroke treated by oral anticoagulation (6.8%) (19). Although NOACs are more advantageous in reducing hemorrhagic stroke and bleeding risk vs. warfarin for AF patients with previous stroke/TIA (21), they are still associated with a high risk of bleeding. Lasek-Bal et al. (22) reported that the incidence of hemorrhagic complications was 12.0% in AF patients with previous stroke after rivaroxaban treatment, which seemed higher than that in our stroke group. Our subgroup analysis indicated that patients with prior stroke did not present with worse outcomes in regards to recurrent thromboembolism and major bleeding risks vs. those without prior stroke post procedure. This implies that LAAC could prevent recurrent stroke and decrease bleeding risk in AF patients, regardless of previous stroke history.

In the present study, despite having a high thromboembolic risk in the stroke group, the observed annual rates of thromboembolism and major bleeding were still as low as 1.3 and 2.5%, respectively after LAAC. Diener et al. reported that the annual rate of stroke/systemic embolism and major bleeding was 4.94% and 5.71% respectively in patients with AF and prior stroke /TIA post-NOAC treatment (23). Moreover, our study showed that the actual annual rates of thromboembolic events were significantly lower *vs*. predicted risks, yielding 77.5, 87.5, and 74.6% in RRR, with NNT 18, 11, and 21 in the overall cohort, stroke group and non-stroke group, respectively. Accordingly, the observed annual rates of major bleeding were also significantly lower than estimated risks, conferring 68.1, 68.8, and 68.6% in RRR, with NNT 20, 18, and 21, respectively. The RRR of thromboembolic and major bleeding events in the overall cohort were in concordance with 2-year follow-up results of RRR of 84 and 70% for thromboembolism and non-procedural major bleeding, respectively, in patients who received concomitant catheter ablation and LAAC procedure (24). A few small-sample studies have investigated the effectiveness of LAAC on thromboembolic and bleeding risks in patients with AF and prior stroke and showed significantly decreased incidence rates of thromboembolic and major bleeding events with respect to estimated risks (12, 25). However, no comparative analyses for the differences of LAAC efficacy in risk reduction of thromboembolism or major bleeding events have been performed between patients with and without prior stroke. Interestingly, our study showed that the percentage of risk reduction for thromboembolic events was significantly greater in stroke group than non-stroke group, whereas the level of risk reduction for major bleeding was similar between groups following LAAC. These findings were quite different from the effects of anticoagulants in patients with AF and prior stroke, in which a few articles reported that the relative effects of NOACs *vs*. warfarin for recurrent thromboembolism in AF patients with prior stroke/TIA were consistent with that of NOACs *vs*. warfarin in AF patients without prior stroke/TIA (26, 27). On this issue, our results expounded more favorable effects of LAAC in patients with previous stroke for risk reduction of thromboembolism. Combined, our research results and prior literature suggest that LAAC may not only decrease the risks of recurrent thromboembolism and major bleeding events in AF patients both with and without prior stroke, but also may provide a greater risk reduction of recurrent thromboembolism for patients with previous stroke compared to those without. It is conceivable that patients with prior stroke may benefit more from LAAC in decreasing stroke recurrence than those without prior stroke. This could be because patients with AF who underwent LAAC exhibited a significantly reduced risk of disabling cerebrovascular outcome after ischemic cerebrovascular events in contrast to oral warfarin treatment (28), and LAAC delivered more quality-adjusted life years as well as more cost-effectiveness relative to NOACs for secondary prevention of stroke in AF patients (29).

Postprocedure DRT formation has attracted great attention as a possible hallmark of thromboembolic events. Some studies demonstrated that the incidence of DRT in patients treated with LAAC ranged from 3.7 to 7.2%, which might be strongly associated with an increased risk of thromboembolic events (30, 31). In a multivariable regression analysis, a history of previous stroke was identified as an independent predictor of DRT (30). However, in the WOLUTION registry, which contains a high proportion of patients with prior stroke/TIA (30.5%) and a positive detection rate of DRT (4.1%), no data was provided on the impact of history of prior stroke/TIA on occurrence of DRT and the correlation between DRT and future thromboembolic risk (32). In our cohort, the incidence rate of DRT was in accordance with those of aforementioned studies and did not differ between patients with and without prior stroke.

In regard to the impact of LAAC on mortality, the overall rate of all-cause death in our cohort reached 15.1%, which was in agreement with other studies which showed that the mortality rate of 2-year follow-up ranged from 9.8 to 20.3% with different ages after implantation of the Amulet Occluder (33). Our subgroup analysis presented that the incidence rate of all-cause mortality or non-cardiovascular mortality was similar between the stroke and non-stroke groups. This conclusion was consistent with recent research results in which no significant differences were observed for mortality between AF patients with and without prior stroke after LAAC (12, 25). In our study, it was noteworthy that the incidence rate of cardiovascular death was significantly lower in the stroke group than the non-stroke group post LAAC. Even after performing PSM analysis to adjust the confounding factors, patients with prior stroke still exhibited a significantly higher cumulative ratio of freedom from cardiovascular death. These findings imply that “the higher the risk, the greater the benefit” for the clinical efficacy of LAAC in AF patients. The reasons for favorable outcomes in cardiovascular mortality from LAAC intervention may be explained in several aspects. First, AF patients with prior stroke were at a higher risk for adverse cardio-cerebrovascular events than those without. A history of previous stroke/TIA was a strong independent predictor for all-cause death, cardiovascular death, and recurrent thromboembolism (16). Second, LAAC may improve mechanical function in left atrium with increased left atrial ejection fraction and left atrial contraction strain (34), and the improvement of left atrial function was significantly associated with a decreased risk of cardiovascular death (35). So, LAAC might play a favorable effect on the risk of cardiovascular mortality. Moreover, previous studies also indicated that LAAC using the WATCHMAN® device could significantly decrease the risks of fatal stroke and cardiovascular/unexplained death compared to warfarin (8). Data from the PROTECT-AF trial and the CAP device registry showed that LAAC could confer net clinical benefit (NCB) of 1.73% per year and 4.97% per year respectively, and especially the NCB of LAAC not only was greater (8.68% per year) in patients with a previous ischemic stroke, but also increased gradually with the increase of CHADS_2_ score (36). Therefore, these results reinforced our research conclusions of greater benefit from LAAC for higher risk patients. In fact, in the PSM cohort with comparable variables between the stroke and non-stroke groups, the difference in the risk of cardiovascular death did not result from different baseline clinical characteristics and postprocedural antithrombotic regimen. Instead, AF patients with previous stroke not only tended to gain, but also might experience greater benefits from LAAC than those without prior stroke. In this regard, a further study with a larger sample size is warranted to evaluate the special subgroup.

In the current study, the overall annualized rate of the combined efficacy endpoints (8.2%) was consistent with those ranging from 5.6 to 11.0% in other studies (9, 37). The annualized rate was similar between the stroke and non-stroke groups.

The main limitations of the study are as follows: (1) this study is a nonrandomized, observational study, so evaluating the effect of LAAC on event rates was limited because of lack of control group; (2) the patient number was relatively small in the overall cohort, and was unequal between groups (74 *vs*. 296), which may be not enough to identify the validity of LAAC on clinical outcomes; (3) the analyses of TEE image and event reporting were performed by investigators with lack of an independent adjudication; (4) as all LAAC procedures were performed by using the WATCHMAN® device, the study findings should be interpreted with caution in other device research.

In summary, LAAC was associated with significantly lower long-term thromboembolic and major bleeding risks compared to predicted risks in the stroke and non-stroke groups. Despite having higher CHA_2_DS_2_-VASc and HAS-BLED scores, patients with prior stroke did not present a worse clinical prognosis compared to those without prior stroke after LAAC. LAAC may provide an increased benefit in the risk reduction of thromboembolism and cardiovascular death in AF patients with vs. without previous stroke. Further research is necessary to evaluate the efficacy of LAAC in this field.

## Data Availability Statement

The original contributions presented in the study are included in the article/supplementary material, further inquiries can be directed to the corresponding author.

## Ethics Statement

The studies involving human participants were reviewed and approved by the Ethics Committee at Helmut-G-Walther Klinikum, Lichtenfels, Germany. The patients/participants provided their written informed consent to participate in this study.

## Author Contributions

MiZ was a major contributor in study design, collecting, analyzing and interpreting the patient data, as well as writing the manuscript. MeZ performed collection and statistical analysis of data, as well as critical revision for the manuscript. CH performed a critical revision for the manuscript. FP performed data analysis and critical revision. NH contributed to data analysis. JW contributed to revision of the manuscript. ZM collected and analyzed the patient data. JY contributed to study design, analysis of data, and critical revision of the manuscript. All authors have read and approved the final manuscript.

## Conflict of Interest

The authors declare that the research was conducted in the absence of any commercial or financial relationships that could be construed as a potential conflict of interest.

## Publisher's Note

All claims expressed in this article are solely those of the authors and do not necessarily represent those of their affiliated organizations, or those of the publisher, the editors and the reviewers. Any product that may be evaluated in this article, or claim that may be made by its manufacturer, is not guaranteed or endorsed by the publisher.
